# Roles of programmed death‐1 and muscle innate lymphoid cell‐derived interleukin 13 in sepsis‐induced intensive care unit‐acquired weakness

**DOI:** 10.1002/jcsm.13548

**Published:** 2024-07-17

**Authors:** Yuichi Akama, Eun Jeong Park, Naoko Satoh‐Takayama, Atsushi Ito, Eiji Kawamoto, Arong Gaowa, Eri Matsuo, Satoshi Oikawa, Masafumi Saito, Shigeaki Inoue, Takayuki Akimoto, Kei Suzuki, Motomu Shimaoka

**Affiliations:** ^1^ Department of Molecular Pathobiology and Cell Adhesion Biology Mie University Graduate School of Medicine Tsu Japan; ^2^ Department of Emergency and Critical Care Medicine Mie University Graduate School of Medicine Tsu Japan; ^3^ Precision Immune Regulation RIKEN Research Unit, Center for Integrative Medical Sciences RIKEN Yokohama Japan; ^4^ Department of Thoracic and Cardiovascular Surgery Mie University Graduate School of Medicine Tsu Japan; ^5^ Faculty of Sport Sciences Waseda University Saitama Japan; ^6^ Department of Disaster and Emergency and Critical Care Medicine Kobe University Graduate School of Medicine Kobe Japan; ^7^ Department of Emergency and Critical Care Medicine Wakayama Medical University Wakayama Japan

**Keywords:** Group 2 innate lymphoid cell (ILC2), ICU‐AW, IL‐13, PD‐1, Sepsis, Slow‐twitch

## Abstract

**Background:**

Intensive care unit‐acquired weakness (ICU‐AW) is a syndrome characterized by a long‐term muscle weakness often observed in sepsis‐surviving patients during the chronic phase. Although ICU‐AW is independently associated with increased mortality, effective therapies have yet to be established. Programmed death‐1 (PD‐1) inhibitors have attracted attention as potential treatments for reversing immune exhaustion in sepsis; however, its impact on ICU‐AW remains to be elucidated. Here, we study how PD‐1 deficiency affects sepsis‐induced skeletal muscle dysfunction in a preclinical sepsis model.

**Methods:**

Chronic sepsis model was developed by treating wild‐type (WT) and PD‐1 knockout (KO) mice with caecal slurry, followed by resuscitation with antibiotics and saline. Mice were euthanized on days 15–17. Body weights, muscle weights, and limb muscle strengths were measured. Interleukin 13 (IL‐13) and PD‐1 expressions were examined by flow cytometry. Messenger RNA (mRNA) expressions of slow‐twitch muscles were measured by reverse transcription and quantitative polymerase chain reaction (RT‐qPCR). In an *in vitro* study, C2C12 myotubes were treated with lipopolysaccharide (LPS) and recombinant IL‐13 followed by gene expression measurements.

**Results:**

WT septic mice exhibited decreased muscle weight (quadriceps, *P* < 0.01; gastrocnemius, *P* < 0.05; and tibialis anterior, *P* < 0.01) and long‐term muscle weakness (*P* < 0.0001), whereas PD‐1 KO septic mice did not exhibit any reduction in muscle weights and strengths. Slow‐twitch specific mRNAs, including myoglobin (*Mb*), troponin I type 1 (*Tnni1*), and myosin heavy chain 7 (*Myh7*) were decreased in WT skeletal muscle (*Mb*, *P* < 0.0001; *Tnni1*, *P* < 0.05; and *Myh7*, *P* < 0.05) after sepsis induction, but mRNA expressions of Tnni1 and Myh7 were increased in PD‐1 KO septic mice (*Mb*, not significant; *Tnni1*, *P* < 0.0001; and *Myh7*, *P* < 0.05). Treatment of C2C12 myotube cells with LPS decreased the expression of slow‐twitch mRNAs, which was restored by IL‐13 (*Mb*, *P* < 0.0001; *Tnni1*, *P* < 0.001; and *Myh7*, *P* < 0.05). IL‐13 production was significantly higher in ILC2s compared to T cells in skeletal muscle (*P* < 0.05). IL‐13‐producing ILC2s in skeletal muscle were examined and found to increase in PD‐1 KO septic mice, compared with WT septic mice (*P* < 0.05). ILC2‐derived IL‐13 was increased by PD‐1 KO septic mice and thought to protect the muscles from experimental ICU‐AW.

**Conclusions:**

Long‐term muscle weakness in experimental ICU‐AW was ameliorated in PD‐1 KO mice. ILC2‐derived IL‐13 production in skeletal muscles was increased in PD‐1 KO mice, thereby suggesting that IL‐13 alleviates muscle weakness during sepsis. This study demonstrates the effects of PD‐1 blockade in preserving muscle strength during sepsis through an increase in ILC2‐derived IL‐13 and may be an attractive therapeutic target for sepsis‐induced ICU‐AW.

## Introduction

Sepsis is a life‐threatening organ dysfunction caused by a dysregulated host immune response to infection and a leading cause of death in ICUs. ICU‐AW is characterized by a long‐term muscle weakness often observed in sepsis‐surviving patients.^1^ ICU‐AW is an important syndrome presenting skeletal muscle dysfunction that persists from acute to chronic phase, thereby resulting in prolonged mechanical ventilation duration in ICU patients and increased medical costs. Importantly, ICU‐AW is independently associated with increased mortality in ICU survivors during the chronic phase, that is, after ICU discharge.^2^ Although recent advances in critical care medicine have improved the survival rates from sepsis in the acute phase,^3^ ICU‐AW remains a significant problem that hinders the improvement of mortality and morbidity of ICU patients during the chronic phase.^2^ Previous studies have shown that the pathogenesis of ICU‐AW is a complex of structural and functional changes in the central nervous system, peripheral nerves, and muscle fibres.^4^ However, there are currently no specific drugs or therapies for ICU‐AW, relying on supportive care and nutritional management to alleviate symptoms. Therefore, it is an urgent task for intensivists and researchers to elucidate the pathophysiological mechanisms of ICU‐AW and to seek specific therapeutic agents and treatment strategies.

Although effective pharmacological treatments for muscle weakness in ICU‐AW caused by sepsis remain to be established, the improvement of immune paralysis in sepsis by the inhibition of PD‐1 in T lymphocytes has attracted much attention as a novel therapeutic option for sepsis. PD‐1 is a check‐point inhibitory molecule expressed on T lymphocytes and innate lymphoid cells (ILCs) and suppresses their effector functions in persistent chronic inflammation in infections as well as in cancers, resulting in T cell exhaustion in the case of T cells. The clinical efficacy of PD‐1 inhibitors to restore exhausted anti‐tumour T‐cell immunity has been established for the treatment of several cancer.^5^ In addition, in recent sepsis studies *in vivo*, PD‐1 inhibitors and PD‐1 knockout have shown improved survival following sepsis.^6^ Based on these findings, clinical trials using PD‐1 inhibitors in septic patients are in progress.^7^ Furthermore, these PD‐1 inhibitors given to septic animal models have a protective effect on various organs, such as the lungs,^8^ small intestine,^6^, ^8^, ^9^ and kidneys^9^; however, the impact of the PD‐1 deficiency on skeletal muscle weakness for sepsis survivors remains largely unknown.

Innate lymphoid cells (ILCs) are novel immune cells responsible for organ homeostasis.^10^ They are mainly tissue‐resident cells that mediate immune responses and getting attention as a novel therapeutic target. ILCs are non‐T, non‐B lymphocytes without antigen receptors and are classified according to the transcription factors required for their development and function and their distinct developmental trajectories, mainly in five groups: Conventional NK, group 1, 2, and 3 ILCs (ILC1s, ILC2s, and ILC3s), and lymphoid‐tissue inducer (LTi) cells.^11^ ILC2s produce CD4^+^ T helper type 2 (Th2) cytokines, including IL‐13, and act as the innate counterparts of Th2 cell effector subsets. ILC2s, as well as T cells, are known to be functionally suppressed by PD‐1‐mediated signals.^12^, ^13^ Recently, novel therapeutic mechanisms mediated by PD‐1 on ILC2 as well as PD‐1 on T cells have been reported in sepsis.^5^, ^14^ However, the effect of PD‐1 inhibition on septic skeletal muscle and localized ILC2 remains to be elucidated.

IL‐13 plays a crucial role in muscle homeostasis and increases mitochondrial activity during exercise.^15^ Therefore, functional analysis of the immune cell in skeletal muscle, such as ILC2, has attracted attention as a way to develop new therapeutic targets for ICU‐AW. Based on these findings, we focused on PD‐1 expressed by ILC2s and IL‐13 locally produced by ILC2s residing in the skeletal muscle. We hypothesized that blocking PD‐1 would prevent muscle weakness through increasing IL‐13 production by ILC2s. A chronic sepsis in WT mice developed skeletal muscle weakness and atrophy; on the other hand, the same model in PD‐1 KO mice preserved skeletal muscle strengths and showed no significant change in skeletal muscle wet weight. Our results point to the potentially important role of IL‐13‐producing skeletal muscular ILC2s and PD‐1‐mediated suppression of the ILC2 in the pathogenesis of sepsis‐induced ICU‐AW.

## Methods

### Mice

Specific pathogen‐free C57BL/6J wild‐type (WT) mice were purchased from Japan SLC (Shizuoka, Japan). Programmed death‐1 knockout (PD‐1 KO) male mice on a C57BL/6 background were kindly provided by Dr. T. Honjo through the Riken BioResource Research Center.^12^ All mice were maintained at the Experimental Animal Facility of Mie University and 12‐ to 17‐week‐old male mice were used in this study. Experimental animal protocols were approved by the Ethics Review Committee for Animal Experimentation of Mie University (approval #: 2019‐41‐1).

### A chronic sepsis model with long‐term muscle weakness

A chronic mouse model of sepsis for the analysis of muscle weakness has already been reported previously.^16^, ^17^ Briefly, caecal slurry (0.1 g/mL) was administered intraperitoneally at the lowest dose that was 100% lethal in the absence of subsequent antibiotics administration or fluids resuscitation. The minimum lethal dose of caecal slurry was 250 μL per mouse based on the survival probability analysis (Figure S1A, B). Mice that did not become severely hypothermic (≤30°C at 12 h) were excluded from this study. Experimental resuscitation to mimic ICU treatment including intraperitoneal injection of antibiotics such as 3 mg (per mouse) of meropenem (Fujifilm Wako Pure Chemical Corporation, Osaka, Japan) and 3 mg (per mouse) of cilastatin (Fujifilm Wako Pure Chemical Corporation) and subcutaneous injection of fluids (sterile physiological saline 0.9%, 700 μL) was performed at 12 h later and repeated every 12 h for seven times.^17^, ^18^ Resuscitation with fluids was discontinued when the murine body temperature was above 35°C. Mice that did not achieve severe hypothermia (≦30°C at 12 h, a clinical sign of sepsis in this model) were excluded from the study. Mice in the control group were injected with 10% glycerol intraperitoneally and subjected to the same resuscitation. Mice were euthanized on days 15 to 17. All mice were kept in a temperature‐controlled room (20–25°C) with a 12 h light–dark cycle and had free access to water and food.

### Body temperature and grip strength test

Internal temperatures were measured by inserting a thermometer into the rectum. A strength meter (DSV‐5N; IMADA, Toyohashi, Japan) was used to measure the grip strength of the four limbs. This test is a widely used and noninvasive method for assessing mouse muscular strength.^19^ For measurements, mice were held by the tail and allowed to grab a grid with their limbs before measurements were taken. The tail was gently pulled so that the torso was parallel to the grid and the maximum grip strength was recorded. As the grip strength method we employed is influenced by behavioural factors in mice, three consecutive measurements were conducted at one‐minute intervals and the average value was designated as the grip strength to minimize behavioural and procedural variabilities. Each value was normalized to the body weight of each mouse. Body temperatures were measured at the same time. Muscle strength was measured immediately before the mice were euthanized.

### RNA isolation and reverse transcription and quantitative polymerase chain reaction

The tibialis anterior (TA) muscle was homogenized using a BioMasher II (Nippi Inc., Tokyo, Japan). Total RNA from homogenized TA muscle, whole blood, or C2C12 myotubes was isolated using Trizol reagent (Thermo Fisher Scientific, Waltham, MA, USA) according to the manufacturer's instructions. Reverse transcription (RT) was performed using a ReverTra Ace qPCR RT Master Mix kit (Toyobo, Osaka, Japan). RNA purity (A_260_/A_280_ ratio: ≥1.8) and concentration were measured using a NanoDrop 2000 (Thermo Fisher Scientific). RT‐qPCR was performed using SYBR Green (Thermo Fisher Scientific) with QuantStudio 3 (Thermo Fisher Scientific). Ribosomal protein, large, P0 (*Rplp0*) and actin beta (*Actb*) were used as endogenous control genes to normalize mRNA levels using the comparative threshold (CT) method. All primer sequences are listed in Table S1.

### Isolation of cells from the skeletal muscle

Cells were isolated from the lower extremities. Muscle tissues were minced into small pieces and incubated with 1 mg/mL collagenase (Fujifilm Wako Pure Chemical Corporation) for 1 h at 37°C. The cell suspension was filtered through a 70 μm and 40 μm nylon mesh (Corning Inc, Corning, NY, USA) and treated with red blood cell (RBC) lysis buffer (BioLegend, San Diego, CA, USA) to remove erythrocytes.

### Histological analysis

Soleus muscles were harvested from WT and PD‐1 KO mice on day 15 of sepsis induction. Samples were fixed in 10% formalin‐neutral buffer solution overnight and transported to the Central Institute for Experimental Animals (CIEA) (Kanagawa, Japan) for histological analysis. Samples were dehydrated, embedded in paraffin, and cut into 3‐μm‐thick sections, which were stained with haematoxylin and eosin (H&E). The mean myofibre cross‐sectional area (CSA) of the soleus muscle from WT and PD‐1 KO was analysed using an ImageJ software. One hundred fifty myofibres from three distinct fields were analysed within each muscle section.

### Flow cytometry

Cell suspensions were incubated with a combination of monoclonal fluorescently conjugated antibodies as follows: CD45.2‐PerCP/Cyanine5.5 (104, BioLegend), CD127‐ PE/Cy7 (A7R34, BioLegend), CD90.2‐APC/Fire 750 (30‐H12, BioLegend), IL‐13‐PE (eBio13A, Thermo Fisher Scientific), PD‐1‐PE (J43, BD Biosciences, San Jose, CA, USA), ST2‐BV421 (U29‐93, BD Biosciences), and CD3ε‐BUV805 (500A2, BD Biosciences). The lineage cocktail for the FITC‐conjugated antibodies was as follows: CD4 (RM4‐4, BioLegend), CD19 (1D3/CD19, BioLegend), CD11b (M1/70, BioLegend), CD11c (N418, BioLegend), TCRβ (H57‐597, BioLegend), TCR γ/δ (GL3, BioLegend), Gr1 (RB6‐8C5, BioLegend), NK1.1 (PK136, BioLegend), FcεRI (MAR‐1, BioLegend), and Ter119 (TER‐119, BioLegend). ILC2s were identified as Lin^−^CD45.2^+^CD90.2^+^ST2^+^ by flow cytometry. To analyse intracellular protein expression, cells were incubated with phorbol 12‐myristate 13‐acetate (PMA) (50 ng/mL) (Nacalai Tesque, Kyoto, Japan) and ionomycin (500 ng/mL) (Nacalai Tesque) in the presence of GolgiStop (BD Biosciences) for 4 h. Cells were analysed on a BD FACSAria fusion flow cytometer (BD Biosciences). Intracellular staining was performed using an intracellular fixation and permeabilization buffer set (Thermo Fisher Scientific). Cell viability was determined using the Zombie Aqua Fixable Viability Kit (BioLegend). Fc block (BD Biosciences) was used to prevent nonspecific antibody binding. Data were analysed using FlowJo software (BD Biosciences).

### Protein extraction and Western blot analysis

Tibialis anterior (TA) skeletal muscles were harvested from WT and PD‐1 KO mice, frozen in liquid nitrogen, and stored at −80°C. The muscles were homogenized using a BioMasher II (Nippi Inc., Tokyo, Japan). Tissues were lysed in ice‐cold Extraction Buffer (Abcam, Cambridge, UK) containing protease inhibitor cocktail (Nacalai Tesque) to extract proteins. Total protein concentrations were measured using Pierce Bicinchoninic Acid (BCA) Protein Assay kit (Thermo Fisher Scientific), according to the manufacturer's instruction. Equal amounts of protein samples (40 μg) were separated on 7.5% sodium dodecyl sulfate–polyacrylamide gels by electrophoresis and transferred to polyvinylidene fluoride (PVDF) membranes. The membranes were blocked with 5% non‐fat dry milk in Tris‐buffered saline supplemented with 0.05% Tween 20 and incubated with mouse anti‐IL‐13α1 (Santa Cruz, Dallas, TX, USA) or rabbit anti‐glyceraldehyde‐3‐phosphate dehydrogenase (GAPDH) (Cell Signaling Technology, Danvers, MA, USA) monoclonal antibodies overnight at 4°C. After washes, the membranes were incubated with horseradish peroxidase conjugated‐anti‐mouse IgG (Cell Signaling Technology) and ‐anti‐rabbit IgG (Cell Signaling Technology), respectively, at room temperature for 1 h. The bands were detected using the ECL kit (Merck, Darmstadt, Germany) and the ImageQuant LAS 4000 mini (Cytiva, Tokyo, Japan).

### Cell culture, maintenance, and differentiation

C2C12 murine skeletal myoblasts (American Type Culture Collection, Manassas, VA, USA) were cultured and maintained in a collagen I‐coated culture plate (Corning, Glendale, AZ, USA) in growth medium containing low glucose DMEM (Nacalai Tesque), 10% Fetal Bovine Serum (Thermo Fisher Scientific), and 1% penicillin–streptomycin (Nacalai Tesque) in an incubator maintained at 37°C with a humidified atmosphere of 5% CO_2_. For differentiation to myotubes, the medium was changed to 2% horse serum (BioWest, Nuaillé, France) in DMEM at 80% confluency. More than 80% of the cells differentiated into myotubes after incubation for 5 days.^20^ Myotubes were treated with lipopolysaccharide (LPS) from *Escherichia coli* O127:B8 (100 ng/mL; Sigma‐Aldrich, St. Louis, MO, USA) for 48 h and recombinant IL‐13 (10 ng/mL; BioLegend) was added for the last 24 h.

### Statistical analysis

Statistical analysis was performed using Prism 9 software (GraphPad, San Diego, CA, USA) and *P* < 0.05 was considered statistically significant. The values for the mean and standard error of the mean (SEM) are presented.

## Results

Severe septic mice were survived by intensive care but still suffered from long‐term sickness.

**Figure 1:**
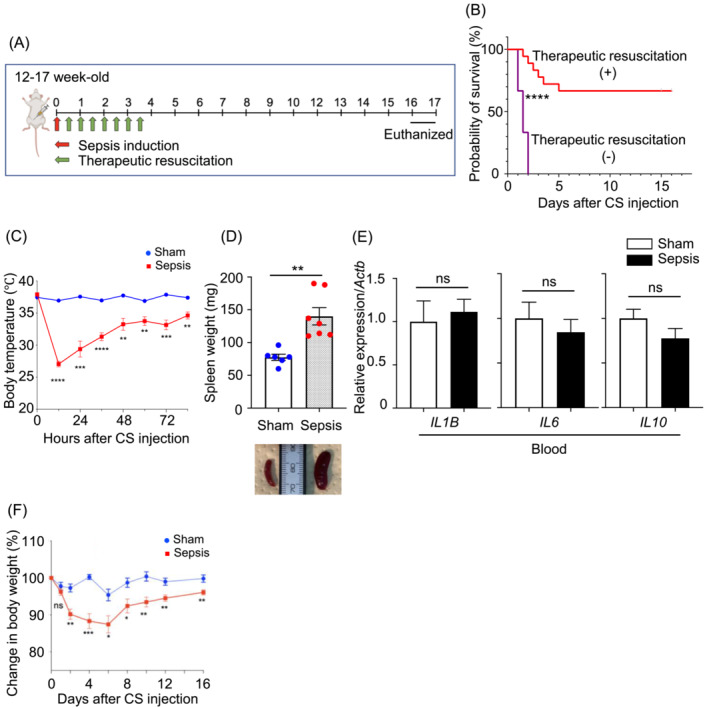
Severe sepsis model showing long‐term body weight loss, but improved blood inflammatory response. (A) Schematic diagram of the protocol which induces long‐term muscle weakness. Sepsis survivors were induced by an intraperitoneal caecal slurry injection (red arrow) and therapeutic resuscitation (green arrows) was initiated 12 h later. Mice were sacrificed on days 15–17 after sepsis induction. (B) Kaplan–Meier analysis revealed a significantly increased survival rate among the sepsis resuscitation treatment group compared with that of the control group in wild‐type (WT) mice (resuscitation treatment, *n* = 18; no resuscitation treatment, *n* = 6). (C) Comparison of temporal changes in body temperatures in sepsis survivors and the sham group in WT mice. Body temperatures were measured from the rectum (sepsis, *n* = 9; control, *n* = 6). (D) Comparison of spleen weights in sepsis survivors and the sham group in WT mice. (E) RT‐qPCR analysis of *IL1B*, *IL6*, and *IL10* expression in whole blood from sepsis survivors and the sham group in WT mice (*n* = 9–12 per group). (F) Comparison of body weight changes in sepsis survivors and the sham group in WT mice. *P* values were determined by a Student's *t*‐test in (C)–(F). Data represent the mean ± SEM. ns, not significant, *P < 0.05, **P < 0.01, ***P < 0.001, ****P < 0.0001.

The tissue‐protective effects of PD‐1 inhibition in sepsis have been reported in the lungs, intestines, or kidneys; however, its role in long‐term skeletal muscle dysfunction has yet to be shown. To address this issue, we used a murine model of long‐term muscle weakness^16^ for both WT and PD‐1 KO mice. Muscle weakness in sepsis was induced by intraperitoneally injecting CS, followed by therapeutic resuscitation to mimic ICU treatment (Figure 1A). Mice receiving the minimal lethal dose of CS with therapeutic resuscitation exhibited approximately 67% survival rate (Figure 1B). All mice in the control group were alive (data not shown). Sepsis survivor mice exhibited hypothermia with body temperatures below 30°C after 12 h of sepsis induction (Figure 1C). The spleens of the sepsis survivor mice were enlarged at the time of the euthanization (Figure 1D), consistent with the presence of systemic inflammation as seen in previous studies using the mice with peritonitis induced by caecal ligation and puncture.^21^ The caecal slurry sepsis model is widely used and has been demonstrated to increase proinflammatory cytokine levels during the acute phase.^22^ Proinflammatory cytokine mRNA expression levels in peripheral blood cells were similar in sepsis and sham mice in the chronic phase (Figure 1E). However, body weights remained significantly reduced during the period of the sepsis (Figure 1F), thereby supporting the idea that this sepsis model recapitulates sepsis survivors with chronic sickness.

### Severe sepsis survivors show persistent skeletal muscle weakness and wet weight reduction in WT but not PD‐1‐deficient mice

**Figure 2:**
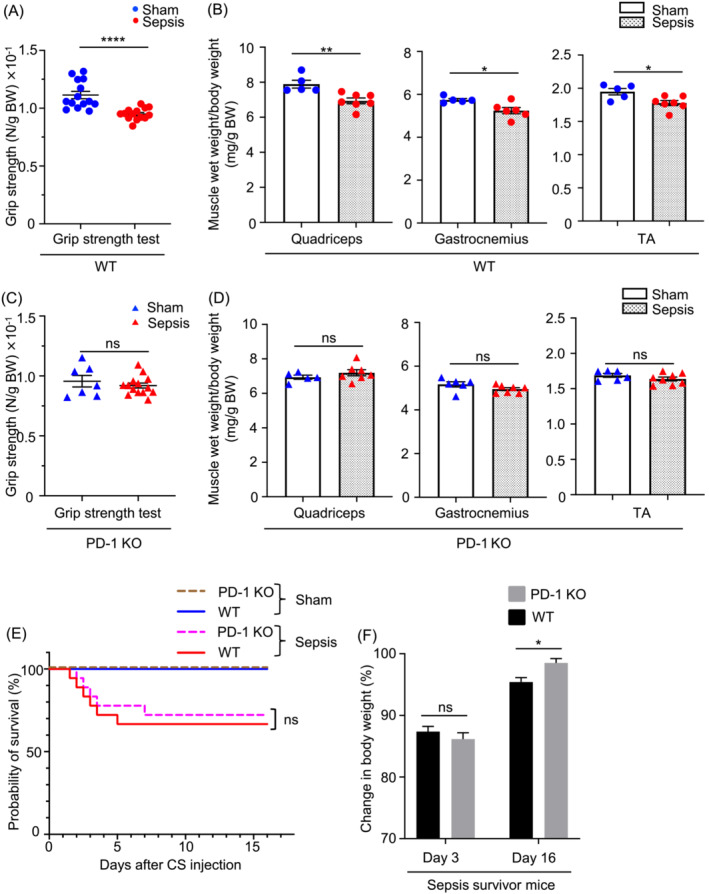
Comparison of muscle strength and wet weight in WT and PD‐1 KO sepsis survivors and the sham control mice. (A) Comparison of muscle force in WT sepsis survivors and the sham control mice. (B) Comparison of muscle wet weight among quadriceps, gastrocnemius, and tibialis anterior (TA) in WT sepsis survivor and the sham control mice. (C) Comparison of muscle force in PD‐1 KO sepsis survivors and the sham control mice. (D) Comparison of muscle wet weight among quadriceps, gastrocnemius, and TA in PD‐1 KO sepsis survivors and the sham control mice. (E) Survival probability in WT and PD‐1 KO sepsis mice was analysed by Kaplan–Meier analysis (WT sepsis, *n* = 18; WT control sham, *n* = 10; PD‐1 KO sepsis, *n* = 18; and PD‐1 KO sham, *n* = 8). (F) Differences in body weight loss improvement between WT and PD‐1 KO mice (*n* = 9 per group). *P* values were determined by a Student's *t*‐test for (A) to (E), and a two‐way ANOVA followed by Sidak's multiple comparison's test for (D). Data represent the mean ± SEM. N, Newton for (A) and (C). BW, body weight. *P < 0.05; **P < 0.01; and ****P < 0.0001.

The body weights (BWs) were different between sepsis survivor and sham mice. Thus, muscle strength was measured and normalized by the BW of each mouse. We found that muscle strength was significantly reduced in the sepsis survivor mice (Figure 2A). In addition, the normalized wet weights of the quadriceps, gastrocnemius, and TA muscles were significantly diminished, suggesting muscle atrophy during the sepsis period (Figure 2B). These results indicate that sepsis survivor mice display muscle weakness and atrophy in a chronic phase of the disease. With the same amount of caecal slurry (250 μL per mouse) used to induce a minimal lethality in WT mice (Figure S1), PD‐1 KO mice were subjected to the induction of the sepsis. There was no significant difference in survival rate between WT and PD‐1 KO mice in sepsis. However, of note, as opposed to WT septic mice, PD‐1 KO septic mice showed neither reduced skeletal muscle strengths nor reduced skeletal muscle wet weights (Figure 2C–E). Intriguingly, the wasting BW in the PD‐1 KO mice was better recovered at the later stage of sepsis, when compared with that in the WT mice (Figure 2F).

### Expression levels of mRNAs associated with slow‐twitch muscle fibres are downregulated in WT sepsis mice, but upregulated in PD‐1‐deficient sepsis mice

**Figure 3:**
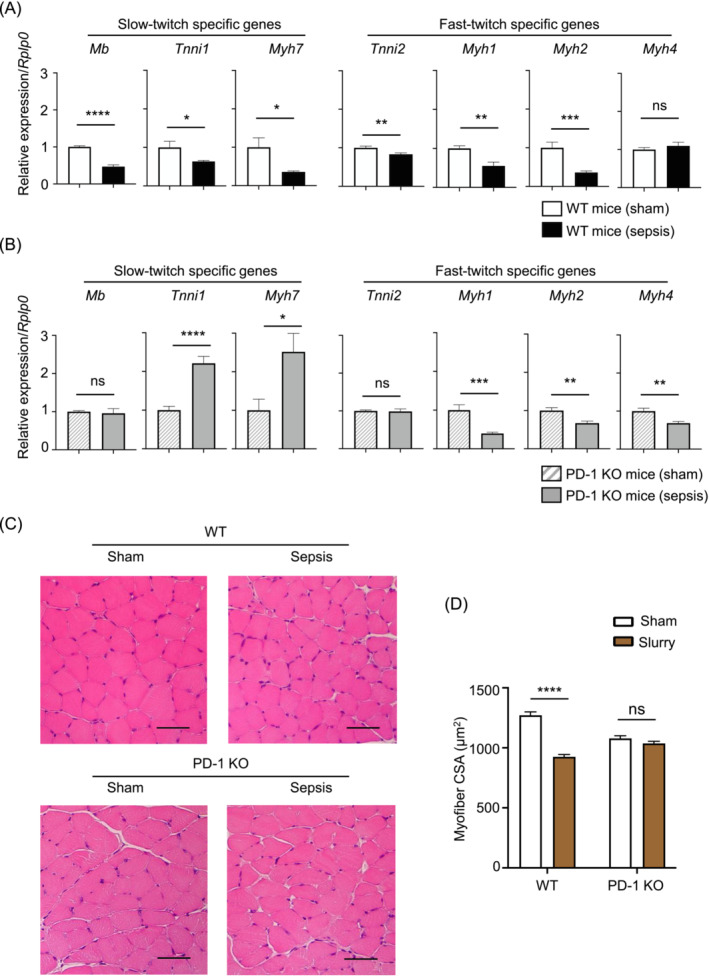
Comparison of the expression of slow‐twitch, myofiber‐specific mRNAs and myofiber CSA in WT and PD‐1 KO mice. (A, B) RT‐qPCR analysis of slow‐twitch‐specific (*Mb*, *Tnni1*, and *Myh7*) and fast‐twitch‐specific gene (*Tnni2*, *Myh1*, *Mhy2*, and *Myh4*) expression in tibialis anterior (TA) muscle tissue (*n* = 11–12 per group). (C) Representative H&E staining images are shown at 400 × magnification (scale bars, 50 μm). (D) Quantified results of myofiber CSA. *P* values were determined by a Student's *t*‐test (A, B) and a two‐way ANOVA followed by Sidak's multiple comparison's test (D). Data represent the mean ± SEM. *P < 0.05; **P < 0.01; ***P < 0.001; ****P < 0.0001; and ns, not significant.

Our results thus far indicated that PD‐1 KO mice showed better capacity in recovering BW loss, compared to WT mice (Figure 2E,F). We thus assumed that muscle weakness of PD‐1 KO mice in sepsis could be relieved by the recovery and synthesis process of relevant proteins compared to that of WT mice in sepsis. Thus, we sought to study the differences in skeletal muscle protein synthesis between WT and PD‐1 KO mice by examining the levels of representative mRNAs. The expression of several fast‐twitch‐specific mRNAs, including *Myh1* and *Myh2*, was significantly downregulated in both WT and PD‐1 KO mice. However, the expression of several slow‐twitch‐specific mRNAs, including *Mb*, *Tnni1*, and *Myh7*, was significantly downregulated in WT, but upregulated in PD‐1 KO mice (Figure 3A,B). To determine a histological basis for the altered gene expressions of the slow‐twitch fibres, we evaluated the myofibril cross‐sectional area (CSA) of the soleus muscle, which is primarily composed of slow‐twitch fibres.^23^ Although there were baseline differences, the CSA of the soleus muscle in WT mice was significantly reduced by sepsis, unlike that in PD‐1 KO mice (Figure 3C,D). Together, these results imply that the downregulation of key proteins associated with slow‐twitch skeletal muscle, such as Mb, Tnni1, and Myh7, may contribute to aggravating muscle weakness and wet weight reduction in WT, compared with PD‐1‐KO, sepsis survivor mice.

### Muscle group 2 innate lymphoid cell‐derived interleukin 13 production is elevated during sepsis in PD‐1 deficient mice

**Figure 4:**
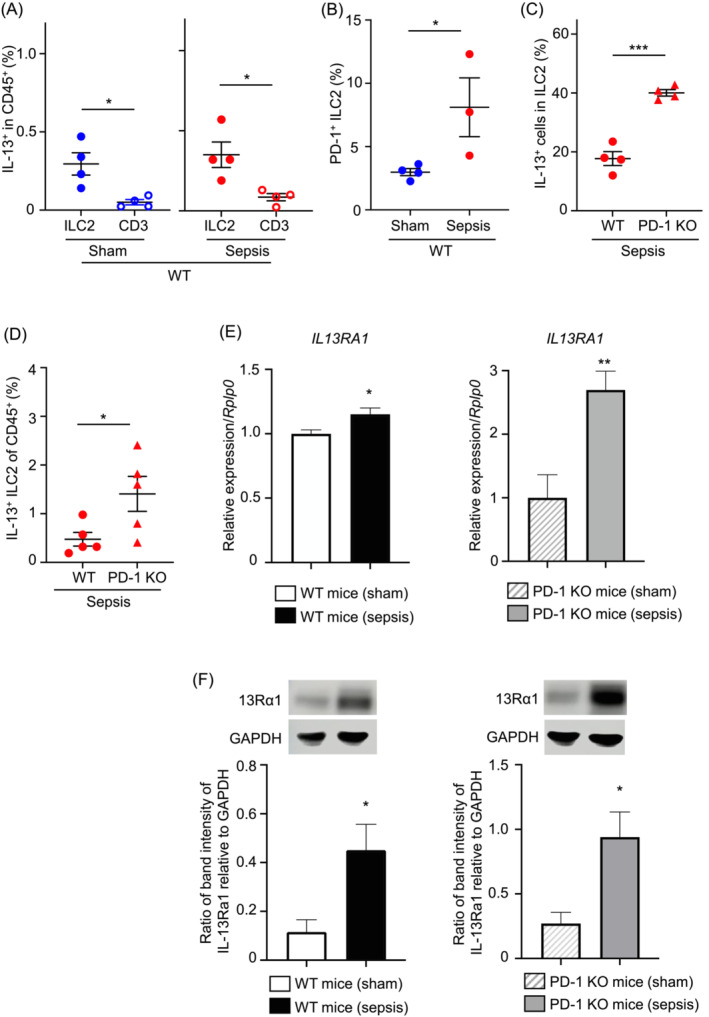
Production of muscle ILC2‐derived IL‐13 is increased in PD‐1 KO mice. (A) Comparison of IL‐13 production in CD3^+^and ILC2s in the skeletal muscle of WT sham and sepsis mice. (B) The percentage of PD‐1^+^ cells in ILC2s of WT mice was increased in the sepsis survivor mice compared to the sham mice. (C, D) The percentages of IL‐13^+^ cells in ILC2s (C) and IL13^+^ ILC2s in CD45^+^ cells (D) in skeletal muscle were increased in PD‐1KO mice compared to those of WT mice. (E) *IL13RA1* expression in WT and PD‐1 KO mice was upregulated in sepsis compared to sham (*n* = 9 per group). (F) Protein levels of IL‐13Rα1 in TA skeletal muscle of WT and PD‐1 KO mice were increased in sepsis compared to sham (*n* = 4–5 mice per group). The ratios of intensities for IL13Rα1 bands to GAPDH bands were obtained using an ImageJ software (F). *P* values were determined by a Student's *t*‐test. Data represent the mean ± SEM. *P < 0.05; **P < 0.01; and ***P < 0.001.

The group 2 innate lymphoid cells (ILC2s) and IL‐13 play an important role in maintaining metabolic homeostasis of skeletal muscles.^15^ Given the abundance of mitochondria in slow muscle fibres, we hypothesized that an increase in intramuscular IL‐13 would contribute to regulating protein synthesis in slow muscle of PD‐1 KO mice following sepsis. We thus sought to determine whether IL‐13 production is increased with PD‐1 deficiency. The CD3^+^ T cells and ILC2s, the cell types that predominantly produce IL‐13, express PD‐1 that signals to suppress the effector functions including IL‐13 production.^12^, ^13^ Therefore, it could be assumed that the capability of CD3^+^ T cells and ILC2s to produce IL‐13 is further increased with PD‐1 deficiency. We found that the frequency of IL‐13^+^ cells in ILC2s was higher than that in T cells of skeletal muscle of WT mice in both sham and sepsis conditions, using flow cytometry analysis (Figures 4A and S2). Moreover, the ILC2s contained more PD‐1^+^ cell population in sepsis, compared to the sham condition of WT mice, while little or no PD‐1^+^ ILC2s existed in PD‐1 KO mice as expected (Figures 4B and S3A,B). We next measured the ratio of IL‐13^+^ cells in intramuscular ILC2s of WT and PD‐1 KO mice in sepsis. The frequency of IL‐13^+^ cells in ILC2s of PD‐1 KO skeletal muscle was higher than that of WT skeletal muscle in the baseline state (Figure S4A). By contrast, both IL‐13^+^ cells in ILC2s and IL‐13^+^ ILC2s in total leukocytes of skeletal muscle in sepsis were significantly augmented with PD‐1 deficiency (Figure 4C,D). In sepsis, PD‐1 KO, but not WT, mice showed a tendency of increasing ILC2‐derived IL‐13 (Figure S4B). Of note, mRNA and protein expression levels of IL‐13Rα1 in both WT and PD‐1 KO skeletal muscle were upregulated by sepsis (Figure 4E,F). The sensitivity of skeletal muscles to IL‐13 was thought to be the most noticeable in the PD‐1 KO sepsis mice. Accordingly, these results suggest that IL‐13 expression is enhanced in ILC2s of skeletal muscle in sepsis and further augmented in the absence of PD‐1 signaling.

### Interleukin 13 protects slow‐twitch‐specific gene expression during sepsis

**Figure 5:**
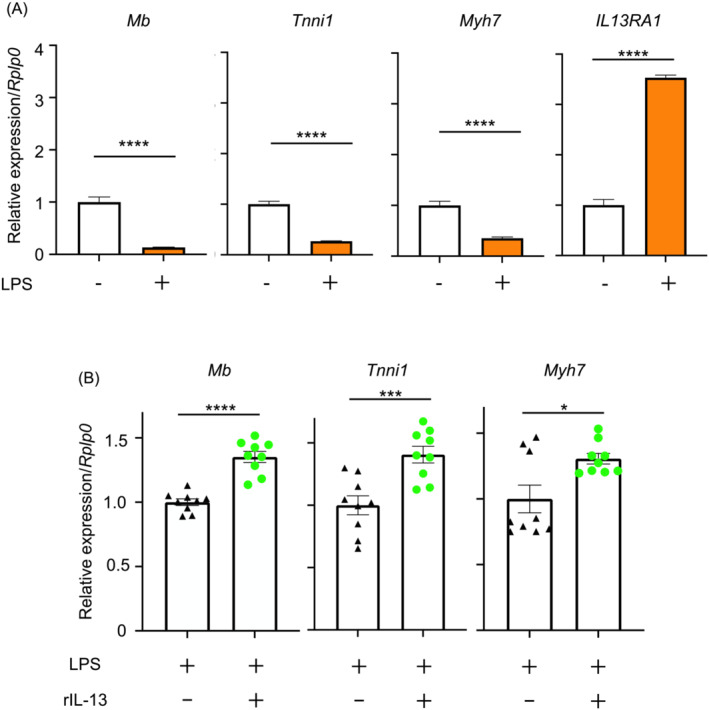
IL‐13 treatment to C2C12 myotubes after LPS stimulation upregulates slow‐twitch‐specific myofibre gene expression. (A) C2C12 myotubes were treated with LPS (100 ng/mL) for 48 h. The expression of *Mb*, *Tnni1*, *Myh7*, and *IL13RA1* was measured by RT‐qPCR analysis. *Rplp0* was used as a housekeeping control gene to normalize mRNA expression levels. (B) C2C12 myotubes were treated with LPS (100 ng/mL) for 48 h and incubated with recombinant IL‐13 (rIL‐13) (10 ng/mL) or mock (PBS) for 24 h. *P* values were determined by a Student's *t*‐test. Data represent the mean ± SEM. *P < 0.05; ***P < 0.001; and ****P < 0.0001.

LPS is one of the most common microbial mediators in sepsis. LPS is also known to involve in skeletal muscle wasting in sepsis and C2C12 myoblast cell line has been often used to test the effects of LPS on myogenesis.^24^ To ask if IL‐13 has any capacity to modify slow‐twitch‐specific gene expression in sepsis, we next employed an *in vitro* model of sepsis‐induced skeletal muscle pathology. Changes in the expression levels of *Mb*, *Tnni1*, and *Myh7* were examined with the LPS‐treated C2C12 myotubes in the presence and absence of recombinant IL‐13 (rIL‐13). Similar to those in skeletal muscles of sepsis mice, expression of *Mb*, *Tnni1*, and *Myh7* mRNAs was downregulated, while that of *IL‐13RA1* was upregulated in LPS‐stimulated C2C12 myotubes (Figure 5A). Intriguingly, expression of *Mb*, *Tnni1*, and *Myh7* was shown to significantly augmented by IL‐13 in the LPS‐treated C2C12 myotubes (Figure 5B). Thus, these results support that IL‐13 possesses a capability in regulating gene expression of the skeletal muscle in sepsis and consequently driving the feasible synthesis of slow‐twitch‐specific proteins.

## Discussion

**Figure 6:**
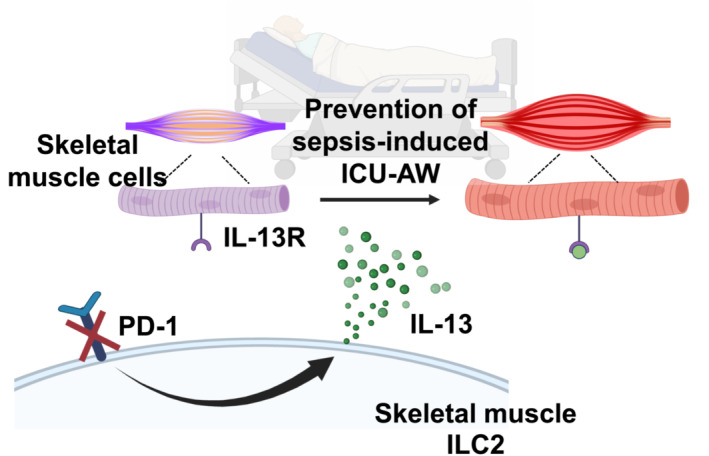
Schematic diagram for the potential role of PD‐1 inhibition and subsequent elevation of ILC2‐derived IL‐13 in intervention of sepsis‐induced ICU‐AW.

In this study, the state of PD‐1 deficiency has been considered to be effective to mitigate skeletal muscle dysfunction and ICU‐AW by analysing muscle strength and slow‐twitch specific gene expressions using a sepsis model. In addition, PD‐1 KO mice showed a significantly increased level of ILC2‐produced IL‐13 and a treatment of IL‐13 to the myotubes enhanced their expressions of slow‐twitch genes in the condition mimicking sepsis. With these notes, we suggest that sepsis‐induced ICU‐AW is possibly prevented by PD‐1 inhibition and subsequent increase in ILC2‐derived IL‐13, as depicted in Figure 6. Therefore, current study would provide an insight into the PD‐1 blockade to be beneficial in, at least partly, preventing sepsis‐induced muscle atrophy and weakness.

PD‐1 inhibitors have been already used for cancer treatment and with proven efficacy and safety for some pathological conditions. The clinical trials are underway and their safety profile in sepsis has been reported.^7^ Our study is an imperative addition to the therapeutic area by newly providing a clinical benefit of the PD‐1 blockade in addition to its previously known effects. Previously, the therapeutic mechanism of PD‐1 inhibition in sepsis focused on the regulation of systemic inflammation through the recovery of exhausted T cells.^14^ Based on our current study, it is suggested that the effects of a PD‐1 blockade on mitigating sepsis can be achieved by a restoration of intramuscular homeostasis contributed by an increased production of ILC2‐derived IL‐13 in the skeletal muscle, in addition to T cell‐mediated regulation of systemic inflammation. PD‐1 inhibition may lead to developing any effective drug for ameliorating ICU‐AW. Regardless of the advances in area of the molecular pathology to date, neither preventive nor therapeutic benefit has been addressed for any drugs including the anabolic steroid oxandrolone, growth hormone, propranolol, immunoglobulin, or glutamine therapy.^25^ Our current study proposes that ILC‐derived IL‐13, which can be increased by a PD‐1 blockade, may contribute to improving any sepsis‐inhibited anabolism, and consequently ameliorating ICU‐AW.

The hallmark of sepsis‐induced muscle weakness is a catabolic state associated with suppressed anabolism.^26^ Our present study provides new insight into PD‐1/ILC2/IL‐13 axis‐ involved anabolic dysregulation in sepsis in aspects of the perspective of immune metabolism. On the other hand, muscle atrophy in sepsis has been known to be primarily affected in fast‐, rather than slow‐twitch, muscle^27^ and to ensue different protein synthesis between both muscles.^28^ But, the effect of PD‐1 deficiency observed in this study was shown to be restricted mainly on slow muscle. Moreover, a possibility that treatment of PD‐1 inhibitor may worse muscle weakness under some conditions cannot be ruled out. PD‐1 inhibition may increase inflammatory cell infiltrates and decrease angiogenesis and regeneration in response to experimental ischemia or contusion injury in muscle.^29^, ^30^ Together, further study on identifying novel functions of PD‐1 in skeletal muscle biology is warranted.

IL‐13 may be a promising factor critical in treating sepsis‐induced muscle weakness. IL‐13 has been shown to prevent diaphragm muscle weakness in an LPS‐induced sepsis model,^31^ supporting the *in vivo* protective effects of this cytokine. IL‐13 has been reported to be anti‐inflammatory. A blockade of IL‐13 led to increased mortality and severer lung injury in a mouse model of CLP‐induced sepsis.^32^ There was another report to find that lung IL‐13 levels were augmented after CLP.^33^ In clinical data, serum IL‐13 levels were markedly elevated in the shock group for the first few days after admission but then diminished to the levels comparable to those in the non‐shock patients.^34^ Interestingly, our present data exhibit a significant increase in mRNA and protein of IL‐13Rα1 in skeletal muscle after severe sepsis (Figure 4E,F). Combined with the previous reports highlighting the importance of IL‐13 in sepsis, IL‐13 is considered to be decreases in sepsis‐induced muscle weakness. ILC2s and T cells are the major sources of IL‐13.^15^ In our study, IL‐13 production in ILC2s was higher than in T cells in both sham and sepsis mice (Figure 4A). PD‐1 KO mice capable of better producing ILC2‐derived IL‐13 preserved the muscle strength in sepsis (Figures 2C and 4C,D). Collectively, the PD‐1/ILC2/IL‐13 axis is supposed to be involved in the the pathogenesis of ICU‐AW by sepsis.

Myoglobin (Mb), troponin I1 (Tnni1), and myosin heavy chain 7 (Myh7) play an important role in regeneration of muscle tissues and are predominantly expressed in slow‐twitch rather than fast‐twitch muscle.^35^, ^36^, ^37^ Mb is thought to play a role in maintenance of muscular homeostasis, because KO mouse model exhibits a decrease in oxidative muscle mass.^35^ In contrast, muscle strengths in myoglobin‐deficient mice are not different from those in WT mice at a steady state,^38^ suggesting that loss‐of‐function for myoglobin can be compensated by other proteins in slow‐twitch or fast‐twitch muscle. However, Mb's function is known for not only oxygen storage, but also regulation of reactive oxygen species in response to intracellular oxygen levels to maintain mitochondrial function.^39^ Thus, it is quite possible that myoglobin deficiency may damage intramuscular metabolism during the post‐sepsis phase. Troponin is a Ca^2+^ regulator for muscle contraction and Tnni1 is an inhibitory subunit of troponin.^36^ Mb is known for its role in muscle contraction and myosin protein and mRNA are reduced in the extremity and trunk muscles in critically ill patients.^40^ Myh7 is the isoform of myosin heavy chain found predominately in slow‐twitch muscle.^37^ Tnni1 and Myh7 directly regulate muscle contraction and decreased expression of these proteins may be involved in muscle weakness in sepsis. In this study, we have focused on analysing mRNA expression levels in TA and considered that the changes in TA expression could consequently modify muscle strengths. It is likely to assume that the change in mRNA expression of slow‐twitch fibres alters the levels and functions of the proteins crucial in tasking muscle strengths, because transcription rates positively correlate with translation efficiency. Our finding indicating that IL‐13 restores the sepsis‐induced decrease in expression of Mb, Tnni1, and Myh7 at the transcriptional level would support the potential roles of IL‐13 in improving or preserving muscle atrophy and weakness in sepsis.

There are some limitations to this study. First, we induced sepsis in PD‐1 KO and WT mice and found no obvious difference in their survival rates. Clinically, ICU‐AW is known to negatively affect life expectancy. The prolonged duration of ventilator withdrawal observed in ICU‐AW affects life expectancy; however, the ventilator was not employed in this model. In this regard, currently used sepsis model may not fully reflect what happens in clinical practice. Thus, it might explain a reason why there was no significant difference in survival despite the alleviation of muscle weakness in the PD‐1 KO mice. The development of an animal model that more accurately recapitulates the prognostic impact of ICU‐AW using a ventilator and the investigation of the effects of PD‐1 inhibition and IL‐13 administration remains challenging. Second, the measurement of food intake amount of mice was not conducted in the sepsis model used in our current study. The muscle weights and strengths of PD‐1 KO mice were maintained at almost normal levels in sepsis (Figure 2C,D). PD‐1 KO mice are known to exhibit the reduced damage of their organs in sepsis.^6^, ^8^, ^9^ Thus, improved muscular weakness in the septic PD‐1 KO mice can be considered to correlate with their increased food intake when compared with that in septic WT mice. But it has been reported that the muscle weakness occurs regardless of food intake in a study that used the same caecal slurry sepsis model to ours.^16^ In addition, our finding showed no significant changes in survival rate following severe sepsis between WT and PD‐1 KO mice (Figure 2E). These observations would support a minimal effect of PD‐1 deficiency on increasing food intake. Third, a majority of our results from the mRNA expression have been obtained by analysing TA, but not gastrocnemius and quadriceps. Sepsis is a systemic disease, and it is reasonable to consider that this syndrome affects all areas in muscles. The sepsis‐induced changes in muscle wet weight were found in not only the TA but also the gastrocnemius and quadriceps of WT mice (Figure 2A,B). Although it is quite likely to assume that the changes in mRNA expression occurred in TA are recapitulated in gastrocnemius and quadriceps, the analysis of all three muscles is required to validate this assumption in the near future. Finally, reduction of skeletal muscle wet weight and CSA were observed at the time point of 2 weeks after caecal slurry injection of WT mice (Figures 2 and 3C,D). These results were quite different from those of the previous study in which the atrophy was evident at day 4 and recovered at 2 weeks after caecal slurry injection.^16^ A possible cause of this discrepancy may arise from the age difference of the mice used and/or the use of different antibiotics for treatment. Our model displayed worse survival in sepsis than previous studies, which might have affected skeletal muscle wet weight. Importantly, we employed the sepsis model in this study that resembles the clinical setting of surviving from severe sepsis for WT and PD‐1 KO mice. Consequently, we could discover that PD‐1 played a role in regulating muscle strengths and weights in sepsis‐surviving mice.

In summary, PD‐1‐deficient mice exhibited a functional and expressional restoration of muscle weakness in sepsis compared to WT mice. Increased production of ILC2‐derived IL‐13 may be involved in the maintenance of muscle strengths and regeneration. Future studies towards preclinical and clinical applications are warranted that aim to investigate the efficacy and side effects of pharmacological PD‐1 inhibition on ameliorating atrophy and dysfunction of skeletal muscle in sepsis.

## Conflict of interest

The authors declare that they have no conflict of interest to disclose with respect to this manuscript.

## Funding

This work was supported by JSPS KAKENHI Grants [Grants‐in‐Aid for Scientific Research, Numbers 21K09069 (Y.A.), 19KK0224 (E.K.), 22K08971 (A.I.), 22K09160 (E.J.P.), and 19KK0196 (M.S.)] and a research grant (Number 2021042945) from the Takeda Science Foundation (Y.A.).

## Supporting information


**Figure S1.** Examination to determine the minimal lethal dose of CS in WT and PD‐1 KO mice. Survival probability in WT (A) and PD‐1 KO (B) sepsis mice was analysed by Kaplan–Meier analysis (WT mice, *n* = 3–5 per group; and PD‐1 KO mice, n = 3–6 per group).
**Figure S2.** The flow cytometry gating strategy and representative dot plots to analyse the IL‐13^+^ cells in ILC2s and CD3^+^ T cells.
**Figure S3.** Comparison of PD‐1^+^ cell population between sham and sepsis of WT and PD‐1 KO mice. (A) The representative dot plots are shown to analyse the PD‐1^+^ cells in skeletal muscle of WT (upper) and PD‐1 KO (lower) mice. (B) The percentages of PD‐1^+^ ILC2s in skeletal muscle of PD‐1 KO mice in the condition of sham and sepsis (*n* = 4 per group). Data represent the mean ± SEM.
**Figure S4.** Production of muscle ILC2‐derived IL‐13 in WT and PD‐1 mice. (A) The percentages of IL‐13^+^ cells in ILC2s in skeletal muscle under normal conditions. (B) Change in the expression ILC2‐derived IL‐13 of WT (*n* = 3–4 per group) and PD‐1 KO (n = 3–4 per group) mice. *P* value (A) was determined by a Student's *t*‐test. Data represent the mean ± SEM.

**Table S1.** List of primer sequences used in RT‐qPCR.
