# Supplementary material for: Roles of programmed death‐1 and muscle innate lymphoid cell‐derived interleukin 13 in sepsis‐induced intensive care unit‐acquired weakness
Source: J Cachexia Sarcopenia Muscle. 2024 Jul 17;15(5):1999–2012. doi: 10.1002/jcsm.13548 (PMC11446709; doi:10.1002/jcsm.13548)
Supplement: Supplementary file 1 — Figure S1. Examination to determine the minimal lethal dose of CS in WT and PD‐1 KO mice. Survival probability in WT (A) and PD‐1 KO (B) sepsis mice was analysed by Kaplan–Meier analysis (WT mice, n = 3–5 per group; and PD‐1 KO mice, n = 3–6 per group). Figure S2. The flow cytometry gating strategy and representative dot plots to analyse the IL‐13+ cells in ILC2s and CD3+ T cells. Figure S3. Comparison of PD‐1+ cell population between sham and sepsis of WT and PD‐1 KO mice. (A) The representative dot plots are shown to analyse the PD‐1+ cells in skeletal muscle of WT (upper) and PD‐1 KO (lower) mice. (B) The percentages of PD‐1+ ILC2s in skeletal muscle of PD‐1 KO mice in the condition of sham and sepsis (n = 4 per group). Data represent the mean ± SEM. Figure S4. Production of muscle ILC2‐derived IL‐13 in WT and PD‐1 mice. (A) The percentages of IL‐13+ cells in ILC2s in skeletal muscle under normal conditions. (B) Change in the expression ILC2‐derived IL‐13 of WT (n = 3–4 per group) and PD‐1 KO (n = 3–4 per group) mice. P value (A) was determined by a Student's t‐test. Data represent the mean ± SEM. [file JCSM-15-1999-s002.pptx]

## Slide 1
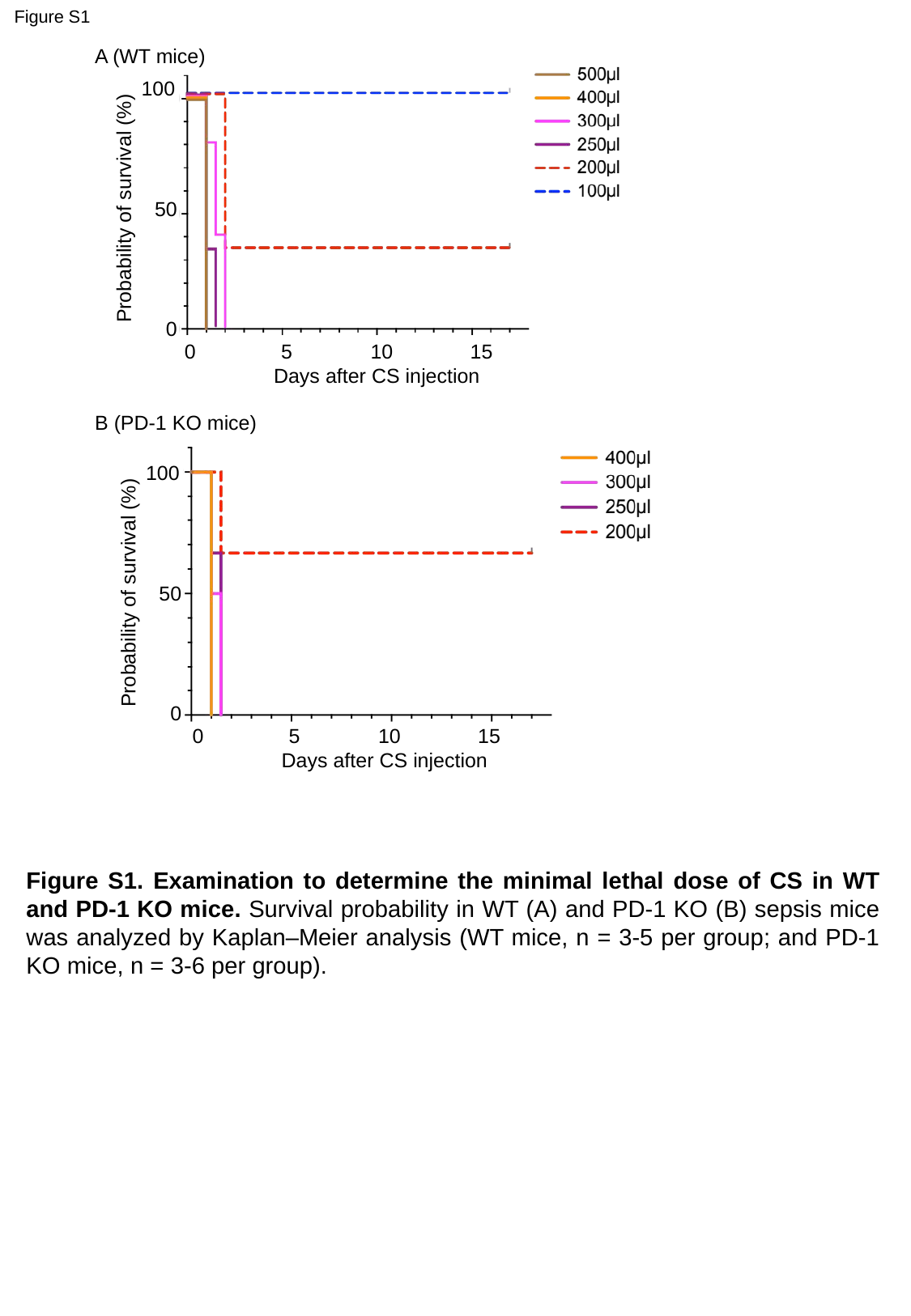

Figure S1
A (WT mice)
100
Probability of survival (%)
50
0
0
5
10
15
Days after CS injection
B (PD-1 KO mice)
100
Probability of survival (%)
50
0
0
5
10
15
Days after CS injection
Figure S1. Examination to determine the minimal lethal dose of CS in WT and PD-1 KO mice. Survival probability in WT (A) and PD-1 KO (B) sepsis mice was analyzed by Kaplan–Meier analysis (WT mice, n = 3-5 per group; and PD-1 KO mice, n = 3-6 per group).

## Slide 2
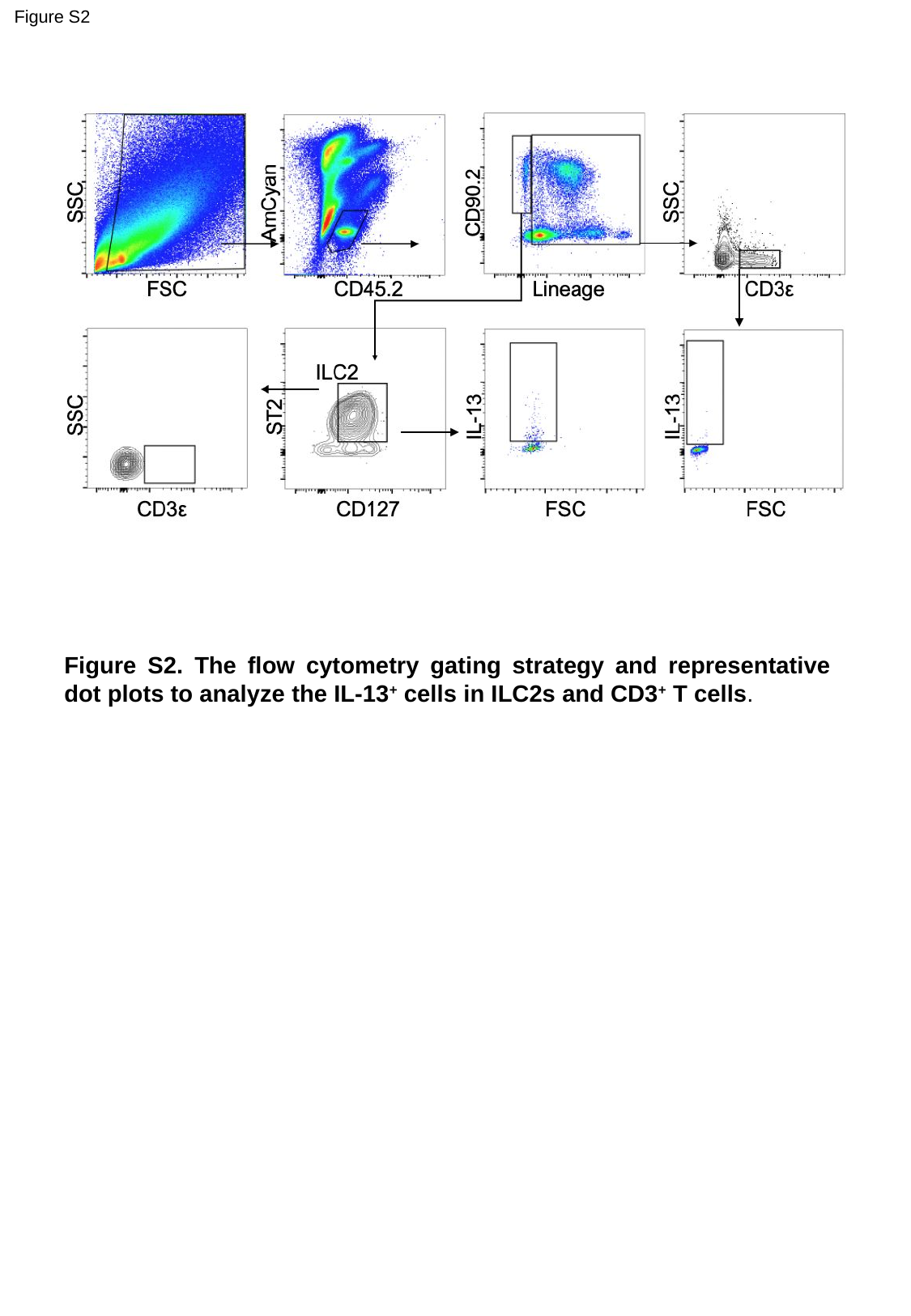

Figure S2
Figure S2. The flow cytometry gating strategy and representative dot plots to analyze the IL-13+ cells in ILC2s and CD3+ T cells.

## Slide 3
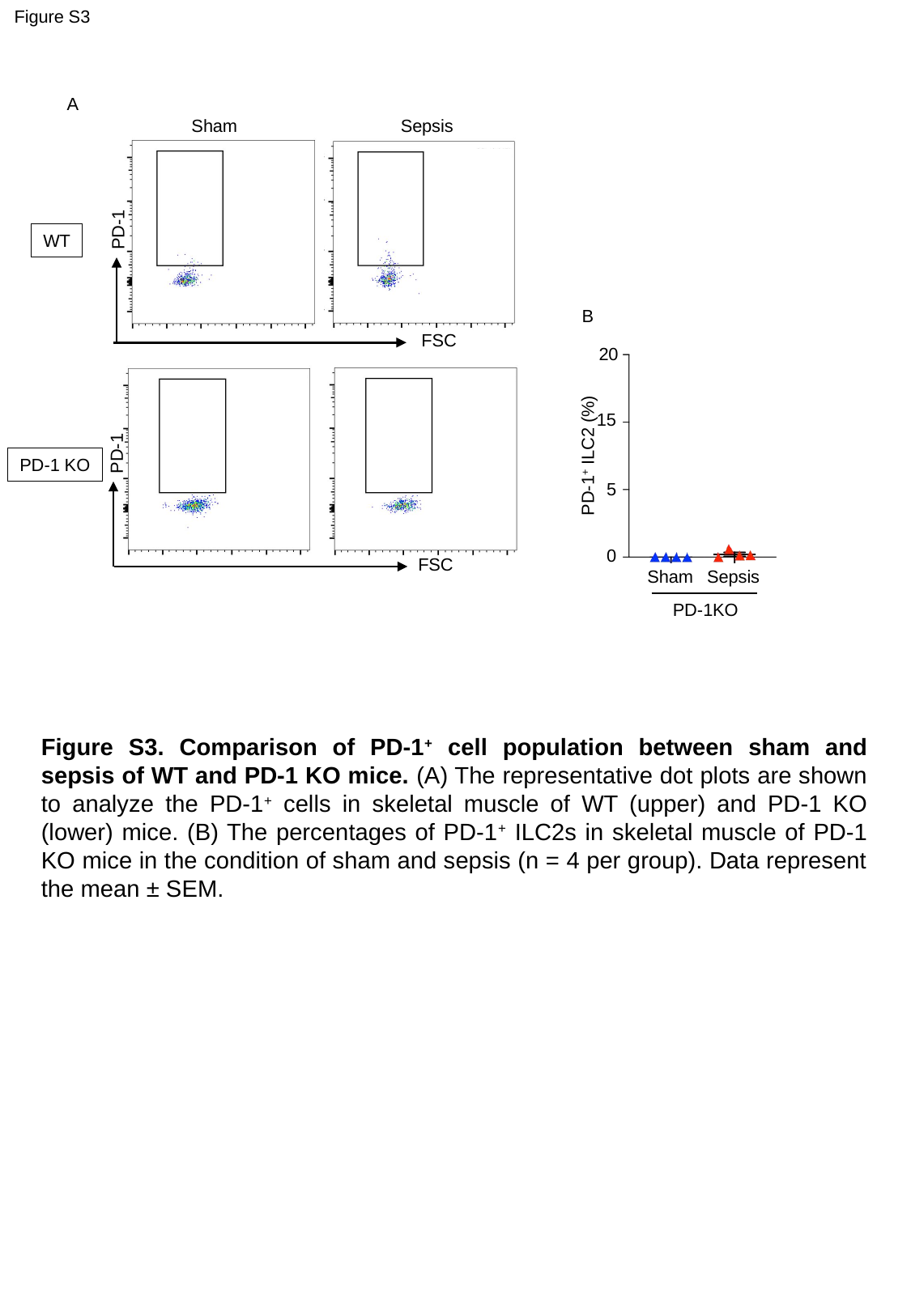

Figure S3
A
Sham
Sepsis
PD-1
WT
B
FSC
20
15
PD-1
PD-1+ ILC2 (%)
PD-1 KO
5
0
FSC
Sham
Sepsis
PD-1KO
Figure S3. Comparison of PD-1+ cell population between sham and sepsis of WT and PD-1 KO mice. (A) The representative dot plots are shown to analyze the PD-1+ cells in skeletal muscle of WT (upper) and PD-1 KO (lower) mice. (B) The percentages of PD-1+ ILC2s in skeletal muscle of PD-1 KO mice in the condition of sham and sepsis (n = 4 per group). Data represent the mean ± SEM.

## Slide 4
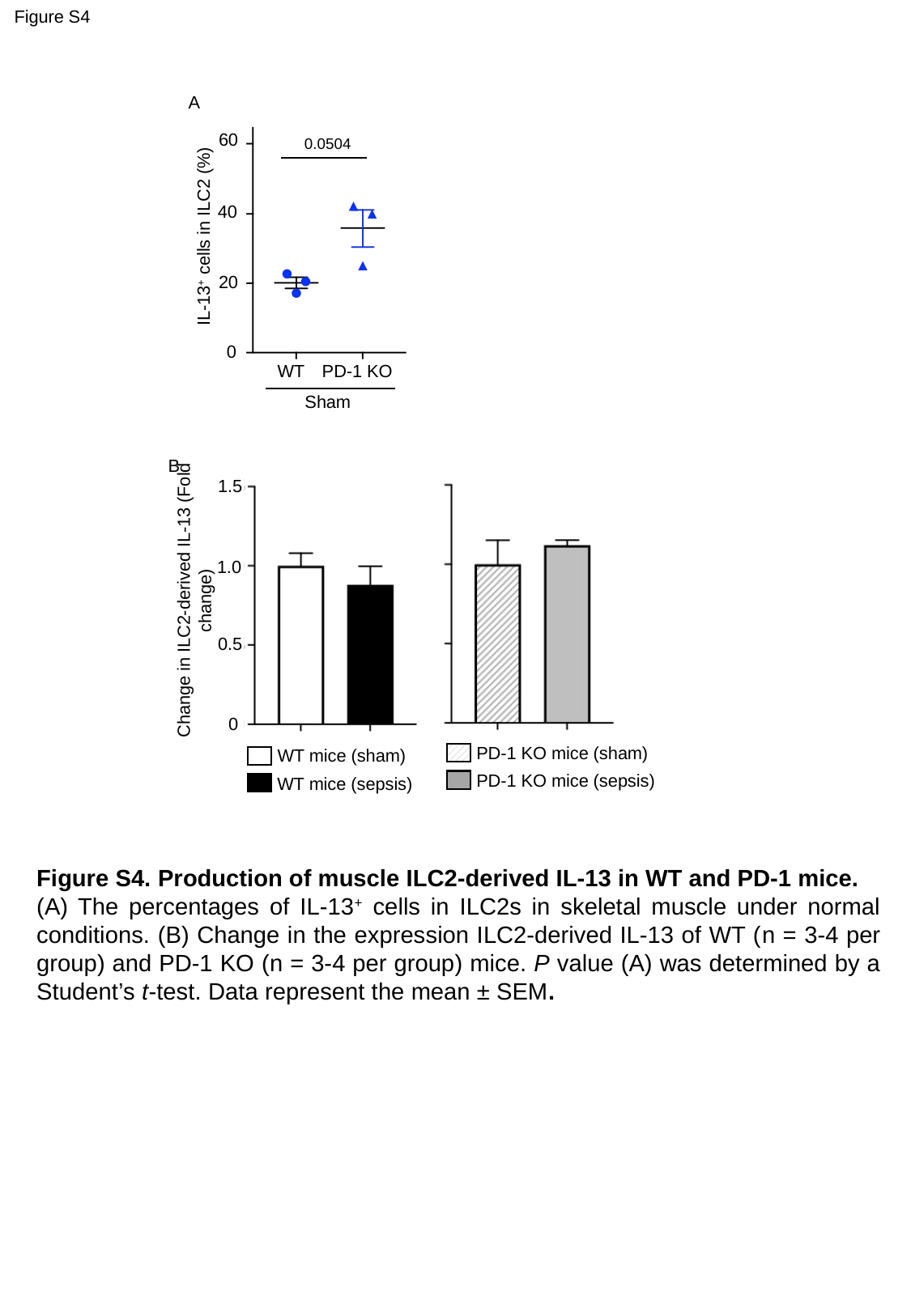

Figure S4
A
60
0.0504
40
IL-13+ cells in ILC2 (%)
20
0
WT
PD-1 KO
Sham
B
1.5
1.0
Change in ILC2-derived IL-13 (Fold change)
0.5
0
PD-1 KO mice (sham)
WT mice (sham)
PD-1 KO mice (sepsis)
WT mice (sepsis)
Figure S4. Production of muscle ILC2-derived IL-13 in WT and PD-1 mice.
(A) The percentages of IL-13+ cells in ILC2s in skeletal muscle under normal conditions. (B) Change in the expression ILC2-derived IL-13 of WT (n = 3-4 per group) and PD-1 KO (n = 3-4 per group) mice. P value (A) was determined by a Student’s t-test. Data represent the mean ± SEM.
